# Incidence and 1-Year Survival of Elderly South Africans Starting Kidney Replacement Therapy

**DOI:** 10.1016/j.ekir.2022.05.030

**Published:** 2022-06-10

**Authors:** Santosh Thapa, Thabiet Jardine, Thaabit Davids, Fergus J. Caskey, Mogamat Razeen Davids

**Affiliations:** 1 Division of Nephrology, Department of Medicine, Stellenbosch University and Tygerberg Hospital, Cape Town, South Africa; 2 South African Renal Registry, Cape Town, South Africa; 3 Population Health Science Institute, University of Bristol, Bristol, United Kingdom

**Keywords:** elderly, kidney failure, kidney replacement therapy, mortality, South Africa, survival analysis

## Introduction

The global prevalence of kidney failure is rising sharply, driven by an increasing prevalence of diabetes, hypertension, infectious diseases, pregnancy-related diseases, environmental toxins, and trauma-related complications. An aging population is another important driver of kidney failure, with the numbers of elderly persons (≥65 years of age) expected to increase rapidly in the coming years.

The South African Renal Registry reports on the treatment of kidney failure with kidney replacement therapy (KRT) in South Africa. The latest report, summarizing 2019 data, gives an overall incidence and prevalence of 15 per million population (pmp) per year and 169 pmp, respectively.^1^ The first data on incident patient survival were published recently.^2^ The 1-year survival rate of 90.4% compares well with the rates reported from better-resourced countries. There have been no reports on the access to treatment and the outcomes in elderly patients in South Africa.

In view of the dearth of data available about elderly patients receiving KRT in low-income and middle-income countries, we analyzed the South African Renal Registry data to provide information on the incidence, treatment modalities and 1-year survival rates of elderly South Africans. Descriptions of the selection of the study population, data collection and analyses are provided in the Supplementary Methods and Supplementary Figure S1.

## Results

Of the 9294 patients who started KRT during the study period (January 2013–September 2018), 1866 (20.1%) were elderly patients (≥65 years of age). During the first 90 days after starting KRT, 61 (3.3%) of the 1866 patients died or stopped treatment. This left 1805 elderly patients alive on day 90. An additional 14 patients recovered kidney function after day 90 and were excluded, leaving 1791 patients to be included in the analysis of 1-year survival. The involvement of participants in this study is illustrated in Supplementary Figure S1.

The incidence of KRT is illustrated in Supplementary Figure S2. Between 301 and 396 elderly patients started treatment annually, an incidence ranging from 110 to 138 pmp per year.

The baseline characteristics of the study population are summarized in Table 1. The median age of the participants was 71.1 years and 58.7% were male. Almost three-fourths of participants were in the 65 to 74 year age group and only 11.1% were older than 80 years. Hemodialysis was the predominant treatment modality and almost all the participants were receiving their treatment in private healthcare facilities.

**Table 1:** Baseline characteristics of incident patients receiving kidney replacement therapy

Variable	All patients (*N =* 9294)		Patients ≥65 (*n =* 1866)		Patients <65 (*n =* 7428)		*P* value
	*n*	%	*n*	%	*n*	%	
Median age (IQR) (yrs)	52.7 (41.3–62.7)		71.1 (68.0–75.5)		48.4(37.9–56.4)		<0.001
Age group (yrs)							
<65	7428	79.9	0	0	7428	100	
65–74	1359	14.6	1359	72.8	0	0	
≥75	507	5.5	507	27.2	0	0	
Male	5553	59.7	1095	58.7	4458	60.0	0.293
Ethnicity							<0.001
Black	5033	54.2	613	32.9	4420	59.50	
Indian/Asian	1204	13.0	385	20.6	819	11.03	
Mixed ancestry	1308	14.1	230	12.3	1078	14.51	
White	1640	17.6	618	33.1	1022	13.76	
Unknown/other	109	1.2	20	1.1	89	1.2	
Primary kidney disease							<0.001
Hypertension	3468	37.3	763	40.9	2705	36.4	
Unknown	2736	29.4	440	23.6	2296	30.9	
Diabetic nephropathy	1821	19.6	511	27.4	1310	17.6	
Glomerular disease	705	7.6	45	2.4	660	8.9	
Other	564	6.1	107	5.7	457	6.2	
Diabetes mellitus							<0.001
Diabetes present	4349	46.8	1171	62.7	3178	42.8	
No diabetes	4266	45.9	572	30.7	3694	49.7	
No data	679	7.3	123	6.6	556	7.5	
HIV serologic status							<0.001
Positive	985	10.6	32	1.7	953	12.8	
Negative	6779	72.9	1569	84.1	5210	70.1	
No data	1530	16.5	265	14.2	1265	17.0	
Hepatitis B serologic status							<0.001
Positive	155	1.7	15	0.8	140	1.9	
Negative or immune	7845	84.4	1619	86.8	6225	83.8	
No data	1294	13.9	232	12.4	1063	14.3	
Hepatitis C serologic status							0.001
Positive	42	0.5	11	0.6	31	0.4	
Negative	7602	81.8	1578	84.6	6024	81.1	
No data	1650	17.8	277	14.8	1373	18.5	
First treatment modality							<0.001
Hemodialysis	7627	82.1	1739	93.2	5888	79.3	
Peritoneal dialysis	1448	15.6	125	6.7	1323	17.8	
Kidney transplant	219	2.4	2	0.1	217	2.9	
Healthcare sector							<0.001
Public	1591	17.1	18	1.0	1573	21.2	
Private	7703	82.9	1848	99.0	5855	78.8	
Province							<0.001
Eastern Cape	1052	11.3	157	8.4	895	12.1	
Free State	583	6.3	79	4.2	504	6.8	
Gauteng	2538	27.3	540	28.9	1998	26.9	
KwaZulu-Natal	2174	23.4	527	28.2	1647	22.2	
Limpopo	394	4.2	81	4.3	313	4.2	
Mpumalanga	329	3.5	40	2.1	289	3.9	
North West	377	4.1	62	3.3	315	4.2	
Northern Cape	159	1.7	17	0.9	142	1.9	
Western Cape	1688	18.2	363	19.5	1325	17.8	

IQR, interquartile range.

Compared with patients younger than 65 years, the cohort of elderly patients included more White patients (33.1% vs. 13.8%, *P* < 0.001) and fewer Black patients (32.9% vs. 59.5%, *P* < 0.001). Elderly patients were more likely to have diabetes (62.7% vs. 42.8%, *P* < 0.001) or a primary kidney disease diagnosis of diabetic nephropathy (27.4% vs. 17.6%, *P* < 0.001). There was a much lower rate of HIV-positive serologic status in patients aged ≥65 years than in patients younger than 65 years (1.7% vs. 12.8%, < 0.001). The use of private sector treatment facilities was much higher among elderly patients than among younger patients (97.9% vs. 78.8%, *P* < 0.001).

**Figure 1:**
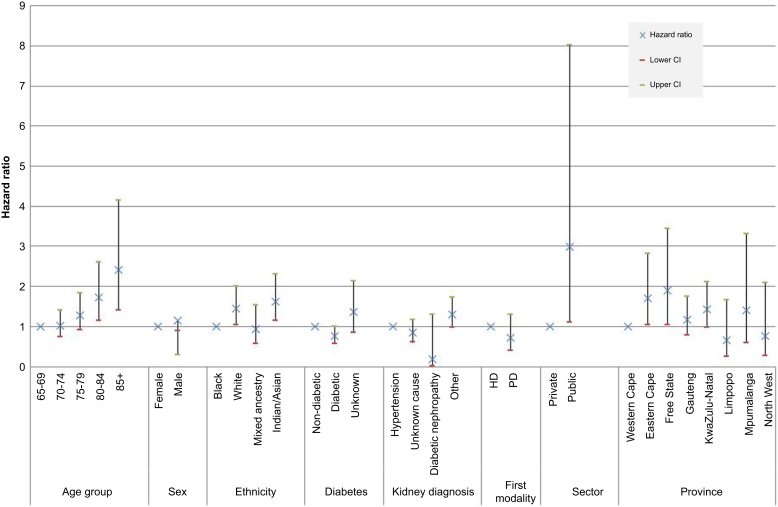
Hazard ratios by multivariable Cox regression for each of the potential risk factors for 1-year survival in elderly incident patients starting kidney replacement therapy. Age group, sex, ethnicity, diabetes mellitus, primary renal diagnosis, first treatment modality, healthcare sector and province of residence were all modeled individually and adjusted for different suites of confounders. HD, hemodialysis; PD, peritoneal dialysis.

A total of 243 patients died within the first year after day 90, yielding a crude 1-year survival rate of 86.4% (95% CI: 84.8%–88.0%). Multivariable analysis of risk factors demonstrated that lower survival was associated with older age, White and Indian ethnicity, and residence in certain provinces (Figure 1 and Supplementary Table S1).

Compared with patients in the 65 to 69 years age group, patients in the 80 to 84 years group had a 72% higher chance of dying (hazard ratio [HR] 1.72, 95% CI 1.14–2.60), whereas those aged 85 and older had even higher mortality (HR 2.42 [1.41–4.14]). Compared with Black patients, White patients had a 44% higher chance of death (HR 1.44 [1.04–2.00]) and Indian/Asian patients had a 62% higher chance of death (HR 1.62 [1.14–2.31]). The patients who were resident in the Eastern Cape and Free State provinces recorded worse survival rates than those in the Western Cape province, with HRs of 1.71 (1.03–2.82) and 1.89 (1.04–3.43), respectively. Neither primary kidney disease nor sex was independently associated with 1-year survival. Patients with diabetes mellitus tended to have a higher survival rate, with a HR of 0.76 (0.57–1.00). The Kaplan-Meier survival curves by age group, with lower survival rates in the older age groups is illustrated in Supplementary Figure S3.

## Discussion

In this first report on the treatment of kidney failure among elderly South Africans, we found low rates of starting KRT, and a 1-year survival rate of 86.4% which compares well with the rates reported from better-resourced countries. Survival was lower with increasing age, among Indian/Asian and White patients, and among those resident in the Eastern Cape and Free State provinces.

The annual incidence of 110 to 138 pmp is much lower than the incidences reported from countries such as the United States (1587 pmp for ages 65–74 years and 1299 pmp for ages ≥75 years) and the UK (310 pmp for ages ≥65 years).^3^^,^^4^ Almost all of our elderly patients were treated in private sector facilities because of the rationing practiced in the resource-constrained public healthcare sector.^5^

Given the high HIV prevalence (13.1%) in the general population,^6^ the HIV prevalence of 1.7% in our cohort is low but that measure must be compared with the HIV prevalence in the South African elderly population of 5.3% for women and 2.0% for men.^7^ In addition, the HIV prevalence among White and Indian/Asian individuals in the general population is about 1% or less,^8^ and our cohort contains a greater proportion of these groups than is found in the general elderly population (33.1% vs. 26.8% and 20.6% vs. 4.2%, respectively).^6^

The 1-year survival rate of 86% in South Africa is comparable with data from better-resourced countries such as the United Kingdom (79%) and the United States (81%).^3^^,^^4^ Nevertheless, it should be noted that the South African cohort is younger than the cohorts from the United Kingdom and United States. Contrary to reports from developed countries, diabetes mellitus was not associated with lower 1-year survival in South Africa. Whereas the reasons for this are unclear, we speculate that patients known with diabetes mellitus are more likely to have been followed up by a specialist physician or nephrologist and to have received good predialysis care and a planned start to KRT. This contrasts with the many patients who present late disease and require urgent dialysis. Late presentation with kidney failure and urgent starts to dialysis are well established as predictors of poor outcomes. It is also possible that some patients with diabetes mellitus who presented late disease and had normal glycated hemoglobin concentrations would have been misclassified as having kidney failure of unknown cause. In addition, the limited duration of follow-up may be a contributing factor to this unexpected finding and studies with longer periods of follow-up are required, especially because the protective effects of early presentation will have most effect in the first year.

We found the survival rate to be lower among Indian/Asian and White patients, and patients who reside in the Eastern Cape and Free State provinces. The reasons for these findings are not clear. Potential contributary factors to the provincial differences include the unequal distribution of treatment centers and human resources for nephrology across the country.^5^^,^^9^

There are some limitations to our study. The sample size of patients reported here is small, is a function of the relatively younger South African population (only 5.6% are ≥65 years of age),^6^ and the rationing of KRT practiced in the public sector, which caters for 85% of the population. Patients who died within 90 days of starting KRT or recovered kidney function were excluded from our study. This common practice ensures that the cohort being reported on has irreversible kidney failure but has the limitation that early mortality is missed. The patients reported on in this study were mostly treated in the private healthcare sector, reflecting access to better resources, including medical insurance. Whereas the results of this study may not appear to be immediately generalizable to other countries, there are many highly relevant lessons because multitiered healthcare systems are present in most countries in Africa (and many low-income countries in other parts of the world).

In conclusion, the incidence of elderly South African patients starting KRT is low, and the treatment of these patients is largely limited to the private healthcare sector. Their 1-year survival is comparable to that reported from better-resourced countries. These results should be considered in planning the equitable delivery of KRT in South Africa.

## Disclosure

All the authors declared no competing interests.

## Supplementary Material

**Supplementary Methods.**

**Supplementary Results.**

**Supplementary References.**

**Figure S1.** Study flow diagram.

**Figure S2.** Incidence of patients starting kidney replacement therapy.

**Figure S3.** Kaplan-Meier survival curves by age group.

**Table S1.** Hazard ratios for death for potential risk factors.
