# Supplementary material for: Incidence and 1-Year Survival of Elderly South Africans Starting Kidney Replacement Therapy
Source: Kidney Int Rep. 2022 Jun 10;7(9):2071–5. doi: 10.1016/j.ekir.2022.05.030 (PMC9458990; doi:10.1016/j.ekir.2022.05.030)
Supplement: Supplementary File (PDF) [file mmc1.pdf]

SUPPLEMENTARY MATERIAL

Incidence and one-year survival of elderly South Africans starting kidney replacement therapy

Santosh Thapa, Thabiet Jardine, Thaabit Davids, Fergus J Caskey, Mogamat Razeen Davids

SUPPLEMENTARY METHODS

The South African Renal Registry (SARR) collects and reports data on adults and children with kidney failure undergoing KRT in the public and private healthcare sectors from all nine South Africa provinces. South Africa has a well-resourced private healthcare sector, which mainly covers the approximately 15% of the population who have medical insurance.[1] The public sector, which serves the 85% without medical insurance, has severe resource constraints, especially as regards the provision of expensive therapies such as dialysis and transplantation.

The methods and definitions used by the SARR are described in the annual reports.[2] In brief, all treatment centres register patients at the start of KRT and submit a small set of data, including the primary kidney disease, diabetes status, serological status for HIV, hepatitis B and hepatitis C, the treatment modality, and the healthcare sector (public or private). Annual updates capture data as at 31 December each year. Any switch in treatment modality and the dates and reasons for stopping treatment are also recorded.

We conducted a prospective cohort study using SARR data to describe the incidence, treatment and one-year survival of elderly South African patients starting KRT. We adopted the World Health Organization's definition of elderly as any person ≥65 years of age.[3]

An incident patient was defined as one who started KRT between 1 January 2013 and 30 September 2018. Due to the requirement to know status at 90 days and the fact that the SARR captures status on 31st December each year, patients beginning KRT in October–December 2018 could not be included. One-year survival was measured from day 90 after the start of KRT. Failure events for the study of survival included death or discontinuation of KRT without recovery of

kidney function. Patients were excluded from the survival analysis if they recovered kidney function during the study period.

Data analysis

Data were exported from the SARR database into Microsoft Excel and analysed using Stata version 16.1 (StataCorp LLC, Texas, USA). Incident rates were reported as patients per million of the elderly population (pmp), with denominators based on the mid-year population estimates of Statistics South Africa (http://www.statssa.gov.za).[4] One-year survival after the initial 90 days was estimated using the Kaplan Meier method and differences between groups assessed using the log-rank test. The association of potential risk factors with survival to one year after 90 days was assessed using Cox proportional hazards regression. Age group, ethnicity, diabetes mellitus, primary kidney disease, first KRT modality, healthcare sector, province of residence and sex were modelled both individually and with adjustment for confounders. Each of these variables was adjusted by different suites of confounders, determined by prior knowledge of whether they met the criteria for confounding (Table S1).[5] There was no significant violation of the proportional hazards assumptions.

Ethical approval was obtained from the Health Research Ethics Committee of Stellenbosch University (reference no. N11/01/028).

SUPPLEMENTARY RESULTS

Figure S1 is a CONSORT diagram displaying the involvement of participants in the study. During the first 90 days after starting KRT, 61 patients (3.3%) died or stopped treatment. Deaths were recorded for 17 patients, with cardiovascular disease the cause in 13 cases. Recovery of kidney function was documented for 12 patients. Reasons for stopping treatment were not always clearly documented but patient/family choice was the reason recorded in five cases and in one case insufficient resources was recorded as the reason.

SUPPLEMENTARY REFERENCES

1. Council for Medical Schemes Annual Report 2019/20. Council for Medical Schemes: Pretoria, South Africa, 2020.

2. Davids MR, Jardine T, Marais N, et al. South African Renal Registry Annual Report 2019. *Afr J Nephrol.* 2021;24:95-106.

3. Kowal P, Dowd JE. Definition of an older person. Proposed working definition of an older person in Africa for the MDS project. World Health Organization: Geneva, 2001.

4. Mid-year population estimates, 2018. Statistics South Africa: Pretoria, 2018.

5. Jager K, Zoccali C, Macleod A, et al. Confounding: what it is and how to deal with it. *Kidney Int.* 2008;73:256-260.

Figure S1. Study flow diagram illustrating the selection of the study population for analysis.

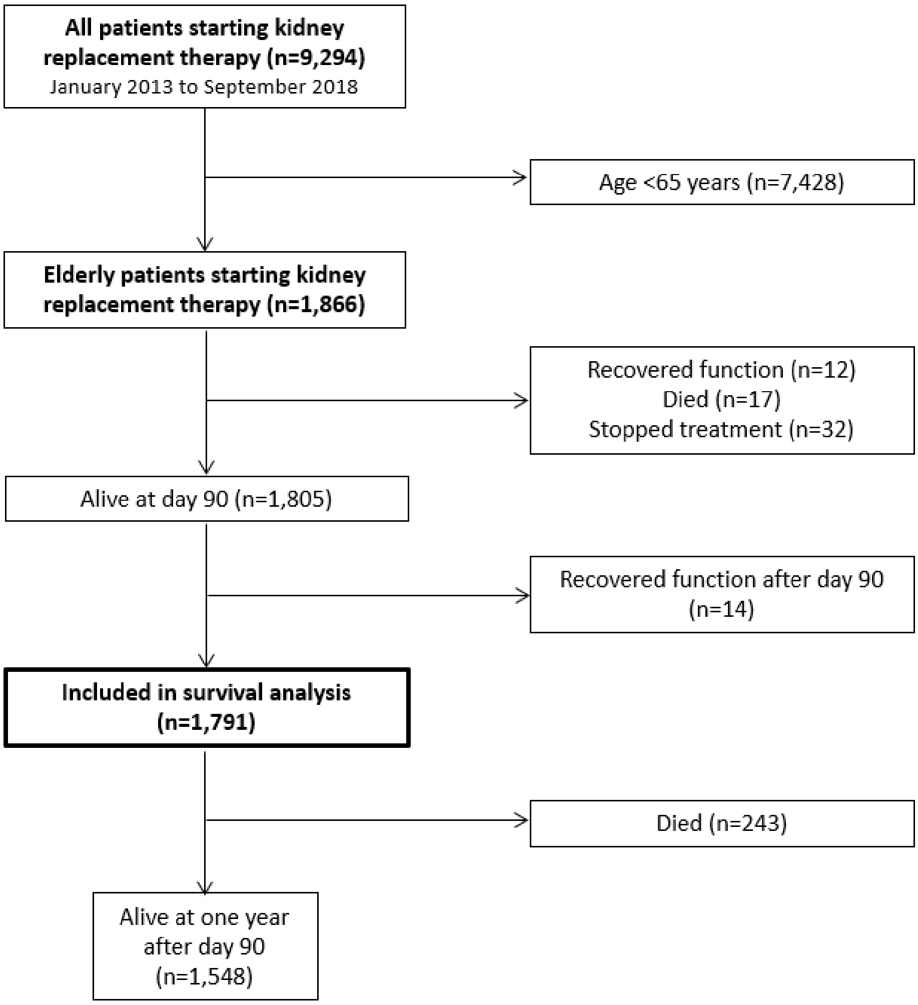

Figure S2. New patients starting kidney replacement therapy each year, and annual incidence of elderly patients, per million population (pmp). The data for 2018 included only the nine months to September of that year and are not presented here.

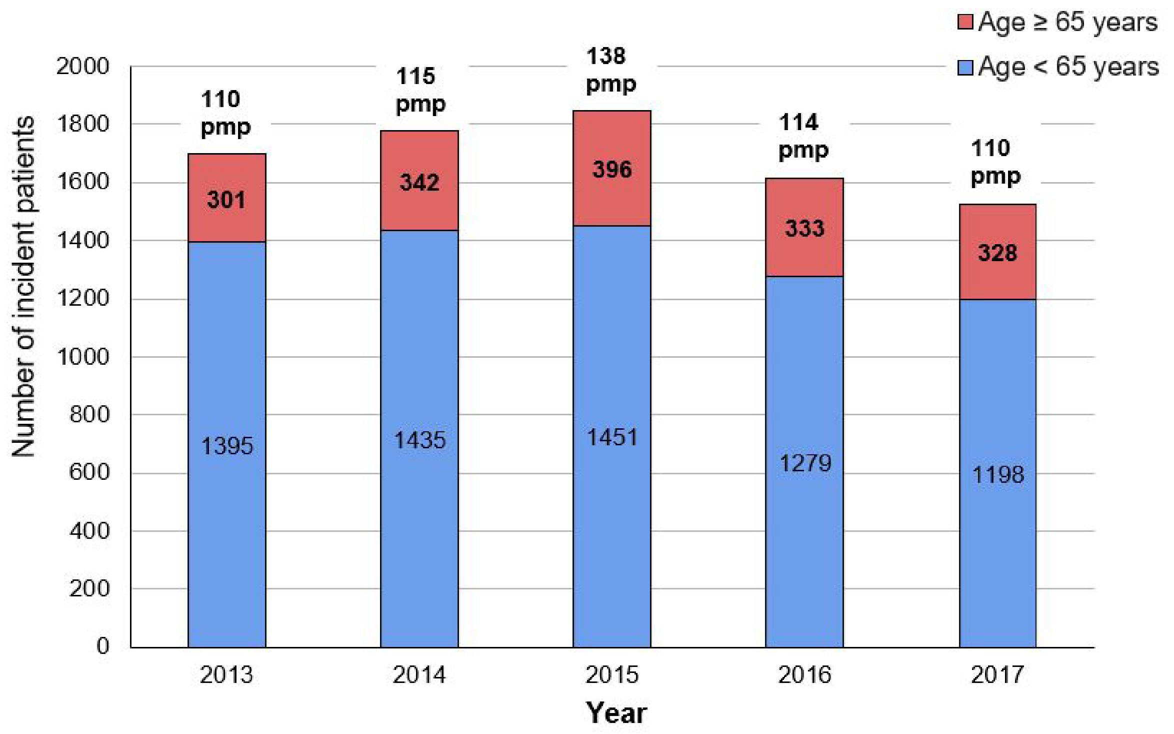

Figure S3. One-year survival from day 90 in elderly incident patients by age group. Log-rank test P = 0.003.

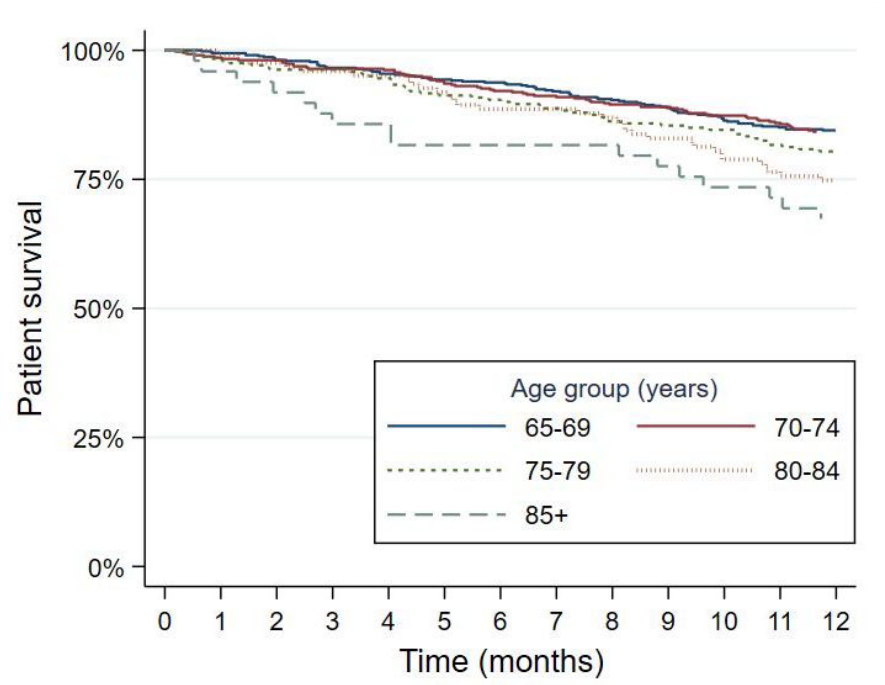

Table S1. Hazard ratios for death for potential risk factors by univariable and multivariable Cox regression. Confounders were selected based on the knowledge that they have an association with both the variable of interest and mortality, without being an effect of the variable of interest nor being present in the causal pathway of mortality.

	**Univariable model**		**Multivariable model**		
**Variable**	**Hazard ratio (95% CI)**	**P value**	**Adjusted for confounder**	**Hazard ratio (95% CI)**	**P value**
**Age group (years)**					
65–69	1		Healthcare sector, ethnicity	1	
70–74	1.03 (0.75–1.43)	0.839		1.02 (0.73–1.41)	0.902
75–79	1.30 (0.91–1.86)	0.151		1.28 (0.90–1.84)	0.170
80–84	1.70 (1.12–2.57)	0.012		1.72 (1.14–2.60)	0.010
85+	2.38 (1.39–4.06)	0.002		2.42 (1.41–4.14)	0.001
**Sex**					
Female	1		Age	1	
Male	1.19 (0.91–1.65)	0.191		1.15 (0.89–1.50)	0.284
**Ethnicity**					
Black	1		Age, healthcare sector	1	
White	1.56 (1.13–2.15)	0.006		1.44 (1.04–2.00)	0.025
Mixed ancestry	0.92 (0.56–1.52)	0.756		0.93 (0.56–1.53)	0.790
Indian/Asian	1.59 (1.12–2.26)	0.009		1.62 (1.14–2.31)	0.007
Unknown/other	No failure events				
**Diabetes mellitus**					
No	1		Age, ethnicity, healthcare sector	1	
Yes	0.71 (0.54–0.94)	0.015		0.76 (0.57–1.00)	0.047
Unknown	1.32 (0.84–2.10)	0.226		1.35 (0.85–2.13)	0.197
**Primary kidney disease**			Age, ethnicity, healthcare sector		
Hypertension	1			1	
Unknown cause	0.80 (0.58–1.11)	0.190		0.84 (0.60–1.16)	0.281
Diabetic nephropathy	0.18 (0.02–1.26)	0.084		0.18 (0.02–1.29)	0.088
Glomerular disease	No failure events				
Other	1.31 (0.98–1.75)	0.069		1.29 (0.97–1.73)	0.083
**First treatment modality**					
Hemodialysis	1		Age, ethnicity, diabetes, primary kidney disease, healthcare sector	1	
Peritoneal dialysis	0.74 (0.41–1.32)	0.302		0.71 (0.39–1.29)	0.264
Kidney transplant	No failure events				

**Healthcare sector**					
Private	1		Age, ethnicity	1	
Public	2.92 (1.09–7.86)	0.033		2.99 (1.11–8.03)	0.030
**Province**					
Western Cape	1		Ethnicity, healthcare sector	1	
Eastern Cape	1.72 (1.05–2.83)	0.032		1.71 (1.03–2.82)	0.036
Free State	1.92 (1.06–3.44)	0.030		1.89 (1.04–3.43)	0.035
Gauteng	1.16 (0.78–1.74)	0.461		1.16 (0.78–1.74)	0.461
KwaZulu-Natal	1.46 (0.99–2.15)	0.056		1.43 (0.97–2.12)	0.070
Limpopo	0.64 (0.25–1.63)	0.348		0.65 (0.25–1.67)	0.374
Mpumalanga	1.40 (0.59–3.30)	0.453		1.40 (0.59–3.32)	0.444
North West	0.74 (0.26–2.08)	0.570		0.75 (0.27–2.09)	0.577
Northern Cape	No failure events				
